# Changes in Tear Proteomic Profile in Ocular Diseases

**DOI:** 10.3390/ijerph192013341

**Published:** 2022-10-16

**Authors:** Mateusz Winiarczyk, Katarzyna Biela, Katarzyna Michalak, Dagmara Winiarczyk, Jerzy Mackiewicz

**Affiliations:** 1 Department of Vitreoretinal Surgery, Medical University of Lublin, 20-059 Lublin, Poland; 2 Department of Ophthalmology, Provincial Hospital of Zamosc, 22-400 Zamosc, Poland; 3 Department of Epizootiology, University of Life Sciences of Lublin, 20-400 Lublin, Poland; 4 Department of Internal Diseases of Small Animals, University of Life Sciences of Lublin, 20-400 Lublin, Poland

**Keywords:** biomarkers, proteomic, MALDI, tear film, ocular biomarker, glaucoma, AMD, cataract, diagnostic

## Abstract

The search for proteomic biomarkers in ocular disease is one of the most important research directions in recent years. Reliable biomarkers can be an immense adjuvant for both diagnostic and therapeutic approaches. There is no more readily available ocular tissue for proteomic analysis than tear film, which makes an interesting target for the biomarker search. Tear film is a complex fluid consisting of a superficial lipid layer, which covers the aqueous-mucous layer. Its complexity makes it a perfect candidate for all the “omics” approaches. Glaucoma, cataract, age-related macular degeneration, and other diseases are commonly thought to have a multifactorial background. Currently, no reliable non-invasive tests are available that would help physicians with screening and further patient management. The aim of the study is to present modern methods of measuring biomarkers in tears, with particular emphasis on spectrometric methods, and to discuss their diagnostic and therapeutic usefulness.

## 1. Introduction

The pathogenesis of various ophthalmic and general diseases is related to the reactions in the body and changes in the microenvironment of the tissues that are affected by the disease, which is reflected in the change in the concentrations of substances involved in these processes. In general diseases, the concentration of desired substance can usually be measured in blood. This examination is widespread and often the basis for diagnosis. Substances labeled for this purpose can be defined as biomarkers-certain molecules that indicate an alteration in normal physiology [1].

Similar relationships can be used in ophthalmology, but the problem is the availability of the diagnostic material. Most studies of the proteome of eye fluids concern the vitreous and aqueous humor. Although widely applied, these tests are invasive and collecting the material can be risky for the patient. In recent years, there have been reports of the possibility of using tears as a source for biomarkers identification.

The aim of the study is to present modern methods of measuring biomarkers in tears and to discuss their diagnostic and therapeutic usefulness.

## 2. Tear Film Collection and Proteomic Analysis

### 2.1. Method of Collecting Tears for Research

There is no clearly defined methodology for tear collection [2]. Moreover, there are several important factors to consider in the study. Dumortier et al. summarized this by presenting the elements necessary to optimize the results, such as tear collection method, sampling time and volume, sample storage and assay conditions [3].

Tears can be collected by direct, indirect and washout methods [4]. Each of these methods has both advantages and limitations.

In the direct method, tears are sucked in through glass microcapillaries. In this way, we obtain basal tears, which is an appropriate material for research [5]; however, the method has its limitations, such as duration, difficulties in obtaining the material and irritation of the eye surface, which may result in the production of reflex tears and, consequently, distort the measurement results [6].

In the indirect method, tears are absorbed onto a specific material, most often Schirmer strips, and then recovered for specific substance determinations [5]. This method is not ideal, as in the process of collecting the material we obtain a mixture of basic and reflex tears, which results in the dilution of the sample and obtaining lower concentrations of the tested substances [2]. Local irritation and vascular permeability may also occur, interfering with the final results [3]. Moreover, the very process of tear collection and processing based on absorption influences the obtained results of chemical analyses [7]. There is also a risk of the contamination of samples with proteins from the surface of the eye [8].

In the flush method, tears are collected similarly to the direct method, but after flooding the eye with physiological saline [9]. The test assumes that the resulting tears will have the same composition as basal tears, but in dilute concentrations [10]. The limitation of this method is the inability to determine substances that are present in tears in low concentrations [2].

The lack of standardization and limitations of commonly used methods results in a continuous attempt to optimize tear collection. For this purpose, new devices are being tested, such as polyester rods [11], polyethersulfone membranes [12], special contact tips [6] or cellulose acetate filters [13]. Although preliminary reports are promising, further research is needed to prove the diagnostic usefulness of the proposed new methods.

Currently, most of the proteomic studies seem to rely on the indirect method of tear collection with the use of Schirmer strips. The indirect method is also better tolerated by patients, making it easier to use in the clinical environment [14]. Most of the studies comparing collection methods did not clearly find any of these superior to another. Many authors have compared indirect and direct methods. Stuchell et al. found the presence of higher concentrations of serum-derived proteins in Schirmer strip samples, which may be due to increased vascular permeability due to conjunctival irritation during this method [8]. Posa et al. pointed out the risk of sample contamination in the indirect method [5]. Markouli et al. assessed that the washout method can only be used for proteins with high concentrations in tears due to the significant dilution of samples [10].

The advantages and disadvantages of each of these methods are shown in Table 1.

### 2.2. Proteomic Analysis

#### 2.2.1. Sample Preparation

The key element of potential protein biomarkers determination is the appropriate sample preparation. In most cases, these procedures include protein extraction or obtainment, purification and eventual precipitation. Both methods of obtaining the tear film and protein extraction should be well suited for the experiment because they strongly affect the quality and composition of acquired samples, especially prior to quantitative analyses. As described in previous work, methods of tear fluid collection are safe and well tolerated by patients, but in terms of physical form, tears collected by capillary sampling are easier to handle and mainly require recovery from a capillary tube by blowing air into the tube and flushing by solvents or centrifugation. There is no need for protein extraction. Nättinen et al. successfully flushed the collecting tubes with 0.5% sodium dodecyl sulphate (SDS) in 50 mM of ammonium bicarbonate supplemented with protease inhibitor cocktail to recover patients’ tears from inside the microcapillary [15]. Other examples of transferring solvents assumes the application of phosphate buffered saline containing 1 mg/mL of BSA (1:25 *v*:*v*) [16], or flushing the tears by instilling 20 µL of 0.9% sodium chloride [17]. Moreover, Kijlstra used phosphate buffer (pH 7.4) containing 4% bovine serum albumin, 1.0 M of sodium chloride and 0.1% Tween 20 as a solvent for capillary-collected tears [4]. Moreover, according to Bachhuber et al., recovering tears from the capillaries is also possible by centrifugation and provides good results [18]. 

In Schirmer strip collection there are many methods for protein extraction, but the simplest method of tear recovery is by centrifugation. Posa et al. placed a Schirmer strip in a 0.5 mL tube that was punctured at the bottom and placed it into a larger tube. Then, the tubes were centrifuged at maximum speed (13,000 rpm) for 5 min. As a result of centrifugal force, fluid was pulled out from the strip material and dripped down into the outer tube [5]. This method is easy and does not lead to any protein modification or structure changes. Elution with tri-distilled water from a strip that was cut into smaller pieces was proposed by Farias et al. In this case, the strip pieces were flooded by 500 µL of MiliQ water and centrifuged for 30 min at 10,000× *g* [19]. There are many publications describing protein extraction from strips by complex mixtures, all of which involve the application of various buffers. Jones et al. extracted protein by 100 mM of ammonium bicarbonate buffer with 50 mM of sodium chloride. The pieces were mixed for 4 h at 25 °C and subsequently centrifuged in a centrifugal filter unit at 7500 rpm for 5 min, after which the strip was removed [20]. An amount of 500 mM of ammonium bicarbonate solution was also used as an elution factor in the manuscript by Huang et al. [21]. Moreover, this process was assisted by 10 min of sonification and heating at 120 °C for 5 min. Green-Church et al. incubated Schirmer strips in approximately 100 μL of 100 mM of ammonium bicarbonate at room temperature for 1 h [22]. For protein extraction, our team used 8 M of urea buffer containing dithiothreitol and CHAPS in the presence of protease inhibitor cocktail. The process lasted for 3 h in 4 °C and was ended by centrifuging (5000× *g* for 20 min) [23,24,25]. Moreover, Ihnatko chose the addition of urea in solubilization buffer for protein extraction [26]. In this case, the composition of the solution was as follows: 20 mM of Tris, 7 M of urea, 2 M of thiourea, 0.1 % CHAPS, 10 mM of 1,4-dithioerythritol, 0.5% ampholyte 3–10 and protease inhibitor cocktail. The strips with collected tears were incubated in the above-mentioned solution for 2 h. Another example of solution that was suitable for protein extraction was a mixture of trifluoroacetic acid and acetonitrile. For this purpose, Powell et al. added solution containing 0.2% TFA:ACN (1:1 *v*:*v*) to a tube with collected tear fluid [27]. The obtained mixture was chilled at −20 °C for 16 h and evaporated using vacuum centrifugation. An extended study about protein extraction was posted in another Green-Church publication [28]. The authors analyzed the relationship between buffer composition and protein recovery. In this case, tears were collected by Schirmer strips and buffers, which were used for extraction, were as follows: (a) 0.9% *w/v* NaCl in phosphate buffer (ph 6.24); (b) 0.9% *w/v* NaCl in phosphate buffer with 0.25% NP-40 protein detergent and 0.25% ABS-14; (c) 100 mM of ammonium bicarbonate; (d) 100 mM of ammonium bicarbonate with 0.25% NP-40 and 0.25% ABS-14; and (e) 40 mM of Tris-HCl, 7 M of urea, 2 M of thiourea, 0.25% NP-40 and 0.25% ABS-14. In each case, extraction lasted for an hour, and the extracted proteins were precipitated with acetone. The biggest protein recovery reaching 36.5 µg was obtained using 100 mM of ammonium bicarbonate with 0.25% NP-40 and 0.25% ABS-14. For protein recovery from the Schirmer strips, Krajcikova performed elution by 100 µL of PBS with a 1% addition of Triton X-100. The tubes with strips and buffer were incubated overnight at 4 °C and centrifuged [29]. 

#### 2.2.2. Protein Purification, Protein Concentration Measurement and Precipitation

The collection technique can strongly influence the protein concentration and profile in tear samples. According to Stuchell and Farias, each technique of tear collection influences the obtained protein amount in the material [8,19]. Regardless of the method chosen, knowing the protein concentration is the key for further analysis. For this purpose, different methods were used, whether direct or indirect. The Bradford method is one of the most popular ones, which is performed in a microplate or standard cuvette format. It is based on the shift in absorbance maximum of Coomassie Brilliant Blue G-250 dye from 465 to 595 nm following binding to denatured proteins in solution [4,5,18,19,30,31,32]. Currently, we observe an increasing participation of direct micro-spectrometric techniques with the A280 program. Changes in absorbance at a 280 nm wavelength are the bases of protein determination in equipment such as NanoDrop or MaestroNano. The application of these methods has many advantages over the traditional Bradford method. Micro-spectrometric equipment requires a small amount of sample (depending on the type from 0.5 to 3 µL) and is quick and safe, as additional sample preparation steps are not required. NanoDrop was used to determine protein level in studies correlated with age-related changes to human tear composition [33] and with a prediction of vernal keratoconjunctivitis reactivation [34]. MaestroNano was applied by our team for the protein measurements of tear fluid in macular edema, due to the neovascular age-related macular degeneration and analysis of tear film obtained from diabetic dogs [24,25]. In terms of protein concentration determination, infrared electromagnetic radiation is also suitable. By measuring amide bonds in protein chains, the modern systems can accurately quantitate an intrinsic component of every protein without relying on amino acid composition, dye-binding properties or reduction-oxidation (redox) potential [35].

From a chemical point of view, tear fluid is a complex mixture containing not only water and proteins, but also organic compounds such as lipids, carbohydrates and inorganic salts, mainly potassium and sodium. Considering that techniques applied in protein separation and identification are extremely sensitive for any kind of interfering substances, it is necessary to purify the samples as thoroughly as possible. For example, salts can disturb the electrophoresis separation, mainly the isoelectric focusing. In this case, focusing will not end until the salt ions reach the end of the strips. Additionally, a significant amount of salts can cause weak focusing at either end of the strip. In term of lipids, their presence can reduce the effectiveness of the detergents as protein-solubilizing agents [36]. To limit potential problems during analysis and reduce the probability of obtaining false-positive or -negative results, the cleaning techniques should be applied. Desalting is possible by performing dialysis, ultrafiltration or precipitation. Removing the low-mass impurities such as salts by ultrafiltration is often conducted by centrifugal filter units. These filters have the abilities of desalting, buffer exchange and protein dialysis. Their application can also cause 30-fold sample concentration. Ultrafiltration 3 kDa cutoff filters were used by Aluru et al. as a purification step before 2D electrophoresis was applied for dry eye syndrome biomarkers determination [37]. Aqrawi et al. described Amicon ultra filters as purifying and concentrating agents during the search for tear biomarkers in primary Sjögren’s syndrome [38]. Protein and peptides concentration and purification can be also performed by ZipTip with a 0.6 or 0.2 µL bed of chromatography media concentrating and purifying femtomoles to the picomoles of protein samples. These chromatographic micro-beds are suitable for sensitive analyses such as MALDI-TOF MS [24,25]. Lipids can by removed in the route of double extraction by ice-cold chloroform:methanol mixture (1:1) at 1200 rpm [17]. Another solution for sample purification as well as precipitation is to apply cleanup kits, which use selective precipitation to remove ionic contaminants such as detergents, lipids and phenolic compounds, improving 2D electrophoresis resolution and reproducibility [24,25].

## 3. Tears as a Source of Information about Eye Diseases

Tear proteome changes can be an important diagnostic clue in numerous eye diseases, such as glaucoma, dry eye disease, diabetic retinopathy, thyroid orbitopathy, pterygium and keratoconus.

### 3.1. Dry Eye Disease (DED) 

Dry eye is a multifactorial disease of the ocular surface that is characterized by a loss of homeostasis of the tear film, and accompanied by ocular symptoms, in which tear film instability and hyperosmolarity, ocular surface inflammation and damage, and neurosensory abnormalities play etiological roles [39]. There are three forms of dry eye disease: aqueous deficient, evaporative due to impaired function of the Meibomian glands, and the mixed form [40]. Most patients suffer from evaporative dry eye [41]. Diagnosis is based on history and clinical symptoms; however, there is no single test sufficient to make a diagnosis [42], and test results are often not correlated with patient symptoms [43]. The search for deviations in the tear analysis is a natural direction in the diagnosis and treatment of dry eye disease.

Numerous studies have shown alterations of the tear proteome in dry eye syndrome. The main tear proteins are lipocalin-1, lactoferrin, lysozyme and the prolactin-induced protein [44], constituting up to 60% of all tear proteins in healthy eyes [45]. All these proteins decreased in dry eye syndrome [46]. This is due to the reduction in the water component of the tear film [47]. A decrease in lactoferrin was observed in patients with mild symptoms of dry eye, which enables diagnosis in patients with normal Schirmer test results [48]. Prolactin-induced protein in dry eye has a diagnostic accuracy of 81%, but a change in the expression of this protein has also been observed in blepharitis, fungal keratitis and keratoconus [49]. This particular protein was determined by using iTRAQ technology combined with nanoLC-nanoESI-MS/MS quantitative proteomics.

A reduction in angiogenin levels was also observed, which correlated with the severity of dry eye syndrome, and thus also with the degree of inflammatory response [50]. This mechanism explains the role of angiogenin as an anti-inflammatory substance. Several studies suggest the diagnostic usefulness of the lacrimal proline-rich protein (LPRP4), which was also decreased [21,37,51]. The technique used to determine the content of this protein was HR-MRM experiments. A UPLC system was coupled to a Q-Orbitrap-MS [21] and two-dimensional difference gel electrophoresis coupled with nano LC-MS/MS [37] or surface-enhanced laser desorption/ionization time-of-flight mass spectrometry (SELDI-TOF-MS) [51]. This protein probably protects the eye by modifying the bacterial flora, but its role is not fully understood [37]. A reduction in the level of the polymeric immunoglobulin receptor (pIgR), a protein that regulates the concentration of secretory immunoglobulin A, appears to be specific to Sjogren’s syndrome [49].

Among proteins with increased expression, the diagnostic utility of lipocalin 2 (LCN2) and alpha enolase was suggested. Lipocalin 2 is an inflammatory response protein that is mainly responsible for the activation of neutrophils, and its increase in tears has been observed in patients with Sjogren’s syndrome. Lipocalin 2 can be easily determined by liquid chromatography connected to a quadrupole precursor with Orbitrap detection, as well as a mass spectrometer [38]. Although alpha enolase appears to be one of the best biomarkers with an accuracy of 85%, its role in the pathogenesis of dry eye syndrome is not entirely clear [47]. Moreover, in this case, mass spectrometry was used, proving iTRAQ technology coupled with 2D-nanoLC-nano-ESI-MS/MS to be a suitable technique. 

In the same paper, the use of marker panels instead of single proteins was also postulated. Diagnostic accuracy was 96% with the use of α-enolase, prolactin induced protein (PIP), lipocalin-1 (Lipo) and calgranulin B (CalB) [47]. It is worth emphasizing that greater differences in the composition of tears were observed in the aqueous deficient compared with the evaporative form of dry eye [45].

One of the most potent proteomic findings in DED was the elevation of matrix metalloproteinase (MMP)-9 expression. It is the only biomarker for dry eye syndrome that has been accepted so far. Inflammation accompanying dry eye syndrome causes an increase in MMP-9 levels > 40 ng/mL. On this basis, a commercially available MMP-9 immunoassay (InflammaDry, Quidel Corporation, San Diego, CA, USA) has become available. However, there are still significant limitations to the usefulness of these kind of tests in clinical practice, as they seem to be dependent on loading volume [52].

### 3.2. Corneal Diseases

Tears are in contact with the cornea and can, therefore, be a source of information about some disorders of this eye structure. Keratoconus and pterygium are examples of such diseases.

Keratoconus is a progressive disease characterized by the thinning and steepening of the central cornea, resulting in irregular astigmatism [53]. The etiology is complex, including genetic, biochemical and environmental factors. Corneal thinning is associated with increased protease activity and decreased protease inhibitors [54]. An optical coherence tomography of the anterior segment of the eye is the best test for the early detection of keratoconus [55]; however, despite advances in diagnostic techniques, diagnosis is often delayed [56].

Differences in protein profiling during corneal diseases were assigned with the help of mass spectrometry. The proteins were first separated by capillary liquid chromatography and later identified by tandem mass spectrometry (nano-LC/MS/MS) [57].

Tear testing in patients with keratoconus can help determine the etiology of the disease, as well as be a part of screening. The tears of patients with keratoconus show, inter alia, an increased expression of metalloproteinase 1 (MMP1) and a decreased tissue metalloproteinase inhibitor (TIMP-1) [57], confirming “the cascade hypothesis of keratoconus”, in which altered levels of individual enzymes lead to cell apoptosis and secondary fibrosis [58]. Additionally, contrary to previous reports, an inflammatory basis of this disease is possible, as evidenced by increased levels of inflammatory molecules in tears, such as metalloproteinase 9 (MMP-9), interleukin 6 and TNF alpha [59].

Pterygium is a degenerative disease in which the conjunctiva grows over the cornea. The etiology is multifactorial, with particular emphasis on the role of UV radiation [60]. UVB radiation causes oxidative stress and, as a consequence, tissue damage and the growth of numerous mediators [61]. Inflammatory cytokines such as Il-1, Il-6, Il-8 and TNF-alpha, as well as angiogenic and fibrogenic factors—especially b-FGF, VEGF, HB-EGF, are associated with the development of pterygium [62]. An examination of the pterygium tissue also showed the overexpression of lipocalin 2 (NGAL), a protein that activates metalloproteinase and, thus, increases the dissolution of the Bowman layer. Protein profiles obtained from diseased and control eyes were also compared using surface-enhanced laser desorption/ionization time-of-flight mass spectrometry (SELDI-TOF-MS) technology. The tears of pterygium patients showed an increased expression of alpha defensins as well as S100 A8 and A9 proteins, which are pro-inflammatory proteins that can be used as a marker to assess the risk of pterygium recurrence after its removal [63].

Although tear proteomic analysis can help us in better understanding the etiopathology of corneal diseases, it should be stressed that they currently have no clinical application in these conditions. 

### 3.3. Glaucoma

Glaucoma is a group of eye diseases, the common feature of which is the characteristic damage to the optic nerve, which results in visual field disturbances [64]. The factor responsible for the damage is increased intraocular pressure. Biomarkers in glaucoma can be used in the early diagnosis, prognosis and evaluation of treatment effectiveness [65,66]. Tears contain proteins derived from the aqueous humor, which are from the uveoscleral outflow pathway [67]. For this reason, they have become the subject of research in glaucoma, although researchers disagree as to whether the protein composition of the aqueous humor corresponds to that of tears [68,69].

MMP-9, a protein involved in angiogenesis, neutrophil inflammatory response and tissue remodeling, may serve as an early marker of glaucomatous changes. Interestingly, its expression decreases in advanced stages of glaucoma, which suggests the exhaustion of the response to tissue degradation [70]. The possible use in diagnostics of endothelin 1, Il-4, Il-12, Il-15, FGF and VEGF [68] has been suggested, since an increase in these substances was observed in the tears of glaucoma patients. The diagnostic utility of CTGF and total tear protein has not been confirmed [71]. Phosphorylated cystatin S (CST4) has also been proposed as a marker differentiating primary and secondary glaucoma. This protein mediates inflammatory responses by releasing interleukin 6. In this study Pieragostino et al. performed a comparative tear proteomic analysis by label-free LC-MS and independently reconfirmed the results by SDS-PAGE and linear MALDI-TOF MS [72]. 

Studies by Pieragostino et al. also emphasize the importance of inflammatory pathways in glaucoma. They confirmed the presence of inflammation-related proteins in the tears of patients with primary open-angle glaucoma and secondary pseudoexfoliative glaucoma. However, it is unclear whether they are related to the disease itself or induced by the therapy. Additionally, there are differences between the inflammatory pathways in primary and secondary glaucoma [72]. Similar conclusions can be drawn from other studies. An increase in tear proinflammatory cytokines such as IL-1B, IL-6, IL-12 and TNFa [73], as well as S100-A8, S100-A9 and mammaglobin B [74]—which were designated by iTRAQ and mass spectrometry—was observed in patients with long-term treatment for glaucoma.

The proteome of the tears in glaucoma patients is also influenced by the type of treatment used. According to a study by Reddy et al., eyes treated with latanoprost overexpressed cytokines were associated with tissue remodeling, while eyes treated with bimatoprost had an increased number of cytokines, which was mainly associated with allergic responses in the eye. Further research in this direction may help minimize the side effects of these drugs [75]. Interferon-γ (IFN-γ), granulocyte-macrophage colony-stimulating factor (GM-CSF) and Interleukin 5 (IL-5) were postulated as prognostic markers of complications after trabeculectomy, and their levels in tears were lowered in the case of complications [69].

### 3.4. Cataract

Cataract remains a major problem worldwide, being one of the major causes of reversible blindness in both developing and developed countries. The etiology and clinical presentation of cataract is diverse, but the most common is the age-related opacification of the natural crystalline lens, which can be operated on with intraocular artificial lens implantation. The studies regarding ongoing proteomic changes during cataract development usually used aqueous humor or the lens itself. In both cases, alterations were mainly shown in the crystallin proteins [76,77,78,79]. Crystallins are one of the most abundant proteins present in human lens. Their main role is to regulate the refractive index of the lens, help with the precipitation of the denaturated proteins and increase cellular tolerance to stress [80]. In this case of crystallins, quantitative proteomics included the iTRAQ methodology [76,77,79], MALDI TOF/TOF mass spectrometry [78]. Currently, there are no reliable studies regarding tear film proteomic changes in the course of cataract formation. Interestingly, cataract group is usually used as a control in the proteomic studies, even though it might create bias in its results. For example, Yao et al. proposed crystalline as one of the potential markers for AMD, while its presence in the aqueous humor might have been a reflection of the cataract formation [81]. For this purpose, a MALDI TOF/TOF MS analysis was performed. A large-scale randomized clinical trial would be of great value in finding potential tear film biomarkers for cataract development.

### 3.5. Tear Film in Retinal Diseases

Although proteomic research in retinal disease is more focused on the vitreous or aqueous humor, tears have become the focus of researchers because of the non-invasive manner of collecting material. By comparing the levels of individual proteins in different eye fluids, tears can be verified as a potential source for specific substances.

Tears have been most widely used in the studies of patients with diabetes mellitus and diabetic retinopathy. Although changes in the tear proteome have been observed in patients with diabetic retinopathy, the mechanism of these changes has not been adequately studied. It seems that this may be influenced by changes in the vascular system and blood circulation in diabetes [82].

In the pathogenesis of diabetic retinopathy, the importance of angiogenic and inflammatory factors is emphasized [83]. This is reflected in tear protein levels. In patients with diabetic retinopathy, a shift in the balance of type-1 T helper and type-2 T helper cytokines towards Th1 was observed in tears. These cytokines determine inflammation and cytotoxicity in the body, while Th2-dependent cytokines have a protective function against these processes [84].

Tears in diabetic retinopathy also show higher concentrations of pro-angiogenic cytokines compared to anti-angiogenic cytokines [84]. Vascular endothelial growth factor (VEGF) has been studied the most, and its relationship with the duration of diabetes and the severity of diabetic retinopathy has been proven [85]. It seems that it may be a useful biomarker in screening studies, where in combination with lipocalin 1 (LCN1) it achieved an accuracy of >80% [86]. Tear VEGF levels were low in patients who responded poorly to anti-VEGF therapy, and it is possible that other treatments should be considered in these patients. LCN-1 has also been considered as an individual diagnostic and therapeutic biomarker for early diabetic retinopathy. For this purpose, Kim et al. used two-dimensional electrophoresis separation coupled with ESI-Q-TOF MS for protein identification [87]. Another protein investigated was tumor necrosis factor alpha (TNF α). It has been proven that the level of this protein increases in PDR compared to NPDR [88], but also increases with the severity of NPDR [89]. These results should be interpreted with caution, bearing in mind that levels of this protein are also higher in ocular surface inflammation [88]. The utility of nerve growth factor (NGF), a protein that modulates angiogenesis and enhances VEGF production, has also been considered. An increase in the level of NGF in PDR was observed, and it correlated with the severity of diabetes expressed by the level of sugar in the blood [90].

Torok et al. proposed the use of tear biomarkers in a model combined with fundus image assessment and micro-aneurism counting. The use of these two methods achieved a sensitivity of 0.93 and a specificity of 0.78 in the diagnosis of early diabetic retinopathy [91]. Perhaps this is the future of screening for diabetic retinopathy.

In age-related macular degeneration (AMD), tears were examined by our team, who found significant differences in tear film composition. In our first attempt we identified eight upregulated proteins in the tear film of AMD patients, but with no quantitative analysis [23]. The second study revealed significant differences with proteins involved in the pathways that are correlated with AMD etiopathogenesis, i.e., inflammation, neovascularization and apoptosis. We have identified three upregulated and eight downregulated proteins, with some of them previously noticed in other tear film proteomic analyses such as retinal dehydrogenase 1 or alpha-enolase [24]. For this, biomarker determination separation by two-dimensional electrophoresis was performed, with protein identification performed by Matrix-Assisted Laser Desorption/Ionization-Time Of Flight (MALDI-TOF) mass spectrometry.

We present up-to-date knowledge concerning the recent findings in proteomic alterations in various ophthalmic diseases in Table 2. The most promising proteomic candidates for biomarkers are summarized in Table 3.

## 4. Conclusions

Tear proteomics holds promise for many clinical applications in the future: it can help to better understand the pathogenesis of certain eye diseases and be used in screening tests, diagnostics and predicting the risk of complications. Finally, it can enable the individualization of therapy and improved monitoring of treatment effectiveness. Commercial dry eye diagnostic tests are already available based on the upregulation of certain proteins. Moreover, proteomics is currently of low significance in regular patient care. Although limited mainly to academic research, with the development of our equipment it may soon be implemented in clinical practice, similar to what happened with genetics. 

## Figures and Tables

**Table 1:** Advantages and disadvantages of tear-collecting methods.

	Direct	Indirect	Washout
**Sample**	Basal tears	Basal + reflex tears	Basal + saline
**Difficulty**	Difficult, long time, small sample size	Easier technique	Easier due to larger sample
**Patient tolerance**	Irritation of the eye surface	Better tolerated, local irritation	Irritation of the eye surface
**Sample quality**	Good	Dilution, risk of contamination	Significant dilution

**Table 2:** Proteomic alterations in tear film of various ocular diseases.

Protein	Dry Eye Disease	Keratoconus	Pterygium	Glaucoma	Diabetic Retinopathy	Age-Related Macular Degeneration
LCN 1	↓				↑	
LCN 2	↑		↑			
Lactoferrin	↓					
Lysozyme	↓					
PIP	↓	↓				
Angiogenin	↓					
LPRP-4	↓					
pIgR	↓					
Alpha enolase	↑					↑
MMP 1		↑				
TIMP- 1		↓				
MMP 9		↑		↓		
Il-6		↑	↑	↑		
TNF alpha		↑	↑	↑	↑	
S100 A8			↑	↑		
S100 A9			↑	↑		
VEGF				↑	↑	
NGF					↑	
Retinal dehydrogenase 1						↑
ABCB1						↑
Annexin A1						↓
Annexin A4						↓
Aldo-keto reductase family 1 member A1						↓
Glutathione S-transferase P						↓
Allograft inflammatory factor 1						↓
Cytospin-A						↓
Short stature homeobox protein 2						↓

**Table 3:** Most promising tear biomarker candidates.

Disease	Substance	Utility	Status	Source
DED	Prolactin-induced protein (PIP), α-enolase	Diagnostic	Suggested	49
	Angiogenin	Severity assessment	Suggested	50
	Metalloproteinase 9 (MMP-9)	Diagnostic Severity assessment	Confirmed	52
	lactoferrin	Early diagnosis	Suggested	48
	pIgR	Diagnosis specific for Sjogren‘s syndrome	Suggested	49
Keratoconus	MMP-1, TIMP-1	Pathophysiology	Confirmed	57
	MMP-9, Il-6, TNFa	Pathophysiology	Confirmed	59
Pterygium	Il-1, Il-6, Il-6, TNFa	Pathophysiology	Confirmed	62
	S100A8, S100A9	Risk of recurrence	Suggested	63
Glaucoma	MMP-9	Early diagnosis	Suggested	70
	Phosphorylated cystatin S (CST4)	Diagnosis (differentiating primary and secondary glaucoma)	Suggested	72
	Granulocyte-macrophage colony-stimulating factor (GM- CSF), interferon-γ (IFN-γ), Interleukin 5 (Il-5)	Prognosis of complications after treatment	Suggested	69
Diabetic Retinopathy	Vascular endothelial growth factor (VEGF) + lipocalin 1 (LCN1)	Screening	Suggested	84, 85, 86

## Data Availability

Not applicable.
